# Early discontinuation of intravenous antimicrobial therapy in pediatric oncology patients with febrile neutropenia

**DOI:** 10.1186/1471-2431-5-10

**Published:** 2005-05-18

**Authors:** Heather Hodgson-Viden, Paul E Grundy, Joan L Robinson

**Affiliations:** 1 Department of Pediatrics Stollery Children's Hospital Edmonton, Alberta, Canada

## Abstract

**Background:**

There are no standard criteria for when to discontinue intravenous antimicrobial therapy (IVAMT) in children with febrile neutropenia (FN), but it is now common to discontinue IVAMT and discharge patients with an absolute neutrophil count (ANC) ≤ 500 /mm^3^. The purpose of this study was to evaluate the outcome of a large cohort of children with FN who had IVAMT discontinued with an ANC ≤ 500 /mm^3^

**Methods:**

A retrospective chart review was completed of patients in the Northern Alberta Children's Cancer Program with FN and no apparent clinical source of fever from June 1, 1997 to July 1, 2002.

**Results:**

Out of a total of 275 patients, 127 (46%) had at least one episode of FN, with FN occurring in patients with sarcomas more commonly than in those with leukemia/ lymphoma and least in those with other solid tumors. In 59 of 276 episodes of FN (21%) patients had a microbiologically defined infection at admission. Of the 217 remaining episodes, 112 of 199 patients (56%) with known neutrophil counts had IVAMT discontinued before their absolute neutrophil count (ANC) reached 500 /mm^3 ^at the discretion of the clinician. Fever recurred in only two of these patients after discharge, and there were no bacterial infections diagnosed after parenteral antibiotics were discontinued.

**Conclusion:**

Even without use of standard criteria for early discharge, clinicians appear to be skilled at selecting children with FN who can safely have IVAMT discontinued with an ANC ≤ 500 /mm^3^.

## Background

Febrile neutropenia (FN) is a common problem in pediatric oncology patients. The majority of patients with FN do not have a microbiologically defined infection (MDI), but those who do are at risk for overwhelming sepsis. It is therefore standard practice to start empiric intravenous (IV) antibiotics if the absolute neutrophil count (ANC) is below 500–1000/mm^3 ^[1]. However, there are no definitive guidelines on the endpoints for stopping antibiotics if a bacterial source is not found for the fever, nor is it clear if the antibiotics need to be given by IV for the entire course. Physicians must balance the potential risk of inadequate antibiotic therapy with the potential risk of antibiotic resistance from overuse of antibiotics, while taking into consideration the emotional, social, and financial costs to hospitalized children and their families.

Management of FN in the Northern Alberta Children's Cancer Program (NACCP) has traditionally consisted of broad spectrum IVAMT (usually tobramycin and piperacillin) until the patient is afebrile, blood cultures are negative at ≥ 48 hours incubation, and the ANC is > 500/mm^3^. However, in recent years, there has been a trend towards discontinuing IVAMT in children who do not have an ANC > 500/mm^3^. Clinicians have used their clinical judgment rather than standard criteria in deciding when to discontinue IVAMT. The objective of this study was to document the clinical course and outcome of FN in the face of this changing management.

## Methods

### Patients

Children ≤ 17 years of age receiving therapy for a primary or recurrent malignancy through the NACCP from June 1, 1997 to July 1, 2002 were enrolled in this study. Inpatient charts were reviewed for these patients at the Stollery Children's Hospital in Edmonton to document the details of all FN episodes. Outpatient charts were also reviewed to determine if the fever recurred after hospital discharge. Fever was defined as a temperature ≥ 38.0 degrees Celsius (°C) measured either at home or in hospital. Neutropenia was defined as an ANC ≤ 500/mm^3^. Children with leukemia who were not yet in remission were excluded from this study as they usually have concomitant complications that prolong hospitalization beyond resolution of the FN.

### Data collection

For each episode of FN, data was compiled from the time of diagnosis of FN until discharge from hospital or death. If cultures done during the first 48 hours of the admission were positive for an organism that would account for the fever, no further data was collected. Fever was not attributed to beta-hemolytic streptococci isolated from a throat swab, as the carrier state is common. Fever was not attributed to toxin producing *Clostridium difficile *in stool, as symptomatic or asymptomatic carriage is common in pediatric oncology patients, yet fever is expected only with severe disease. Organisms isolated from blood cultures were considered contaminants if common skin organisms were isolated from a single blood culture.

For all admissions for FN where an infectious etiology was not identified from cultures done within 48 hours of admission, data was collected on the choice and duration of IVAMT (including outpatient antibiotics), use of oral antibiotics for empiric treatment of FN, use of systemic anti-fungal agents, microbiologic laboratory results, absolute phagocytic count (APC), ANC, absolute monocyte count (AMC), length of stay in hospital attributable to FN, duration of fever and recurrence of fever during or after the admission. A recurrent fever was defined as a new fever that occurred after the patient had been afebrile for ≥ 24 hours, and before they had an ANC > 500/mm^3^.

### Statistical analysis

Data was analyzed using SAS (Cary, NC; Version 8.2). Descriptive statistics were used to summarize the patient cohort.

The Health Research Ethics Board of the University of Alberta approved this study.

## Results

### Patients and episodes of FN

From June 1, 1997 to July 1, 2002, the NACCP treated 275 children. Table 1 is a summary of the number of episodes of FN by diagnosis. All but a small number of children with neuroblastoma and central nervous system tumors received chemotherapy. Of the 275 patients, 148 (54%) were never admitted for FN, and the other 127 patients had 276 admissions for FN. The highest incidence of admissions for FN was in patients with osteogenic and Ewing's sarcoma, with the lowest incidence being in patients with Wilms tumor and central nervous system tumors.

**Table 1:** Episodes of febrile neutropenia by diagnosis in 275 pediatric oncology patients.

Diagnosis	Number of patients (%)	Number of patients with FN episodes (%)	Mean number of episodes per patient	Range of number of episodes per patient
Osteogenic sarcoma	8 (3%)	7 (87%)	3.13	0–7
Ewings sarcoma	5 (2%)	4 (80%)	2.20	0–5
Rhabdomyosarcoma	15 (5%)	11 (79%)	1.93	0–6
Hepatoblastoma	9 (3%)	5 (56%)	1.22	0–5
Acute myeloid leukemia (AML)	16 (6%)	7 (44%)	1.00	0–5
Neuroblastoma	23 (8%)	11 (48%)	0.83	0–5
Non-Hodgkin's lymphoma	24 (9%)	14 (58%)	0.75	0–4
Acute lymphoblastic leukemia (ALL)	85 (31%)	41 (48%)	0.73	0–7
Others *	22 (8%)	9 (41%)	0.41	0–3
Hodgkin's lymphoma	25 (9%)	8 (32%)	0.36	0–5
Central nervous system tumors	26 (9%)	7 (27%)	0.23	0–3
Wilms tumor	17 (6%)	3 (18%)	0.12	0–1
Total	275	127 (46%)		

Children with leukemia not in remission were excluded. All but a small number of children with neuroblastoma and central nervous system tumors received chemotherapy.

n = number of patients

* adrenocortical carcinoma, epithelial tumor, juvenile myelomonocytic leukemia, Langerhans cell histiocytosis, small cell tumor, teratoma

### Microbiologically documented infections

Cultures taken within 48 hours of admission detected MDI that could account for the fever in 59 episodes of FN (21%) in 45 children. Sixteen of these episodes were attributed to viral infections (9 respiratory syncytial virus, 5 parainfluenza, 1 influenza A and 1 influenza B) and 44 episodes were attributed to bacterial infections (5 urinary tract infections, 33 episodes of bacteremia, and 5 other bacterial infections). There were no systemic fungal infections diagnosed in the 276 admissions for FN. Table 2 shows the incidence of infection according to age group.

**Table 2:** Incidence of proven bacterial or viral infections.

Type of infection	Age 0–1 year n = 40	Age 2–4 years n = 91	Age ≥ 5 years n = 145
Viral infection	2 (5%)	6 (7%)	7 (5%)
Bacterial infection	9 (23%)	17 (19%)	18 (12%)
Cultures negative	29 (73%)	68 (75%)	120 (83%)

The table shows the number of proven bacterial or viral infections diagnosed on cultures done within 48 hours of admission during 276 admissions for FN in pediatric oncology patients.

n = number of episodes of FN in this age range

Cultures taken more than 48 hours after admission detected a MDI in eight episodes of FN, but the organisms isolated may not have been the cause of the original fever. These infections were documented at a median of 6.5 days (range 3 – 29 days) after admission to hospital. There were four viral infections (influenza A, parainfluenza, herpes stomatitis (2 episodes)) and four episodes of bacteremia (*Salmonella enteritidis*, viridans streptococci, coagulase-negative staphylococci (2 episodes)).

### Duration of fever

Fever was documented only prior to the hospitalization and not after admission in 72 of the 217 episodes of FN without proven bacterial or viral infection at admission. However, for the 145 episodes where fever was documented in hospital, the median duration of the initial fever was 2 days (range 1 – 33 days). There were 39 episodes of FN in which the fever recurred prior to hospital discharge and persisted for a median of 3 days (range 2 – 19 days).

### Antimicrobial therapy

For two episodes of FN, IVAMT was not initiated, but the reason for this was not clear. For the remaining 215 episodes, the most frequently prescribed IV IVAMT was piperacillin/tobramycin (75% of episodes). The median duration of IVAMT was 5 days (range 1 to 28 days), and outpatient IVAMT were not used. Oral antibiotic therapy was used in 17 episodes prior to hospital discharge and in another six episodes at discharge, but always to treat a specific infection rather than as empiric therapy for FN.

In 16 episodes of FN, patients were on fluconazole prophylaxis at the time of admission. A systemic antifungal agent was added during the hospitalization in another 19 episodes (fluconazole in 15, amphotericin B in 3 and ketoconazole in 1). The anti-fungal was added a median of 2 days after admission (range 1–10 days).

### Clinical course and outcome

The median length of stay in hospital for all FN episodes was 5 days (range 1 to 33 days). Almost all patients were discharged on the day their IVAMT was discontinued.

Phagocytic cell counts at the time of discontinuation of IVAMT are shown in Table 3 (not available in 77 episodes). The median ANC at the time of discontinuation of IVAMT was 400/mm^3^, and in 28/199 episodes (14%), IVAMT were discontinued before the ANC rose to 100/mm^3^. In 13/199 episodes (7%), IVAMT was discontinued when the absolute phagocyte count (APC) was < 100/mm^3^.

**Table 3:** Hematologic parameters when antibiotics were discontinued and at discharge in pediatric oncology patients with FN with negative cultures at admission.

	Median	Range	Number (%) < 100	Number (%) 100–500	Number (%) > 500
ANC when antibiotics discontinued (n = 199)	400	0–34900	28 (14%)	84 (42%)	87 (44%)
Monocyte count when antibiotics discontinued (n = 199)	400	0–4400	20 (10%)	117 (59%)	62 (31%)
APC when antibiotics discontinued (n = 199)	800	0–39300	13 (7%)	56 (28%)	129 (65%)
ANC at discharge (n = 194)	500	0–42200			

The table shows the hematologic parameters when antibiotics were discontinued and at discharge in pediatric oncology patients with FN with negative cultures at admission. There were 217 episodes of FN analyzed in the study, but not all patients had bloodwork done when antibiotics were discontinued or at discharge.

n = number of episodes of FN

ANC – absolute neutrophil count

APC – absolute phagocyte count (ANC + monocyte count)

Two patients died during their admission for FN. One patient was receiving palliative care. The other patient died from complications of graft versus host disease. None of the patients required readmission because of clinical deterioration, suspected infection or recurrent fever prior to their neutropenia resolving.

With four other episodes of FN, a fever recurred after the patient was discharged, but prior to their next chemotherapy treatment. With two of these four episodes, the patient was not neutropenic at the time of the fever and cultures were not taken. For the other two episodes, the patients were neutropenic when the fever recurred (ANC had been 0 and 200 at discharge), but all cultures were negative and the children were not readmitted.

## Discussion

This study was a review of 276 admissions for FN in 275 pediatric oncology patients over a 5-year period. Almost half the patients had at least one hospital admission for FN, with the maximum number of admissions being seven. Admissions for FN occurred most commonly in patients with sarcomas, less so in those with leukemia or lymphoma and least in those with other solid tumors. However, episodes of FN in children with leukemia who were not yet in remission were excluded since FN in this clinical setting often occurs in patients that have other complicating factors.

Management of FN is a controversial issue. It is clear that empiric treatment of FN with IVAMT (with addition of antifungal agents if fever persists) decreases mortality [1]. This is because many of these patients have bacteremia, and septic shock is a common outcome in neutropenic patients who are bacteremic with virulent organisms such as coliforms, *Staphylococcus aureus*, and even some typically non-virulent organisms such as viridans streptococci. However, cultures are never 100% sensitive, not all body sites are accessible to culture, and it is possible that some infections in immunocompromised patients are due to organisms that are cell-wall deficient so are not detected in routine cultures [2]. A study using high-resolution computed tomography of the chest in adults with FN revealed a high incidence of pulmonary infiltrates that were not evident on plain films [3], so it is possible that at least some patients with FN have a bacterial pneumonia that is not diagnosed. Therefore, IV or oral antibiotics are often continued even if all cultures at presentation are negative. However, it is now clear that the majority of adults and children with FN, negative bacterial cultures, and without clinical evidence of bacterial infection do not have a partially-treated or occult bacterial infection, and IVAMT can be discontinued prior to the ANC reaching 500/mm^3 ^[1].

In the current study, an MDI was identified by cultures on admission in 21% of episodes (16% bacterial, 6% viral), and based on cultures done more than 48 hours after admission in 2.8% of episodes (1.4% bacterial, 1.4% viral). There was no mortality and minimal morbidity attributable to infection in the other 208 hospital admissions for FN. Almost all episodes were managed with IVAMT initially, but in 56% of episodes, IVAMT was discontinued when the ANC was ≤ 500/mm^3^. Early discontinuation of IVAMT was based on the judgment of the clinician rather than on standard criteria. Fever recurred in only 4/112 (4%) of these episodes, and the fever was not due to bacterial infection and did not result in readmission in any of these four episodes. This suggests that the clinicians were able to select patients who could safely have IVAMT discontinued and be discharged when the ANC was ≤ 500/mm^3^.

The main potential risk of limiting the duration of IVAMT is recurrence of a partially treated bacterial infection. Benefits of limiting the duration of antibiotic therapy include decreasing the duration of hospitalization, decreasing the risk of antibiotic-related toxicity, and possibly decreasing the risk of secondary infection with organisms that are resistant to the antibiotics that were used for empiric treatment of FN. In the current study, there was no evidence of recurrence of undiagnosed partially treated bacterial infections after discontinuation of antibiotics. With regard to secondary bacterial infections, a bacterial infection was diagnosed based on cultures done more than 48 hours after admission in only 4/276 (1.4%) of admissions, and all four patients were still on IVAMT. In the only published pediatric study where 73 patients were randomized to continue or discontinue antibiotics before the ANC reached 500/mm^3^, the rate of documented or probable secondary bacterial infections was 8% in the group who continued vs. 6% in the group who discontinued antibiotics [4]. Clearly it would require a very large study to demonstrate if early discontinuation of antibiotics in selected patients can decrease the risk of secondary bacterial infection.

There were 21 pediatric studies up to 2002 looking at the outcomes of children with FN who had all antibiotics discontinued or were changed to oral antibiotics with ANC ≤ 500 /mm^3 ^[5]. In general, outcomes were good if patients had evidence of marrow recovery at the time of discontinuation of IVAMT. However, there is a need for specific criteria in determining when to stop empiric IVAMT in patients with FN. Risk factors for bacteremia or other serious bacterial infections that have been validated and would be identifiable soon after admission include:

1) recent chemotherapy with a platelet count <50,000/mm^3 ^[6]

2) relapsed leukemia [6]

3) hypotension [6]

4) CRP ≥ 90 mg/L [6]

5) absolute monocyte count (AMC) < 100 [7-9]

6) peak temperature ≥ 39°C [8,9]

Even these risk factors are not consistent between studies, which could be because the definitions of significant bacterial infections and the populations studied varied [8]. Other factors that have been reported to increase the risk of bacterial infection such as young age [5], high number of previous FN episodes [10], marrow involvement [11], presence of a central venous catheter [11], anticipated neutropenia > 7 days [5,12], presence of co-morbidities [12] or recent stem-cell transplant [5] require validation.

Risk factors for bacterial infections after discontinuation of IVAMT in children with FN are even less well defined. Most studies to date had insufficient power to look at the occurrence of bacterial infections after discontinuation of IVAMT, and used recurrence of fever as an endpoint [5]. As mentioned previously, there is a correlation between the absence of marrow recovery and adverse outcome after discontinuation of IVAMT [1,5], but it is not clear if the APC, the ANC, the AMC, the platelet count, or some combination of these parameters should be analyzed to determine that marrow recovery is occurring. In our study, none of the patients had bacterial infections after IVAMT was discontinued and only four patients with recurrent fever, so we were not able to analyze risk factors. Our study suggests that children selected for early discontinuation of IVAMT by experienced clinicians are likely to have an excellent outcome. However, further prospective studies are needed to determine whether using objective criteria (such as an AMC > 100) in addition to clinical judgment increases the number of children who can safely have their IVAMT discontinued while they have a low ANC.

If IVAMT is discontinued with an ANC ≤ 500/mm^3^, there is no consensus as to whether oral antibiotics should be substituted until the ANC reaches 500/mm^3^. All antibiotics were discontinued with an ANC ≤ 500/mm^3^in at least some patients in 10 of 21 previous pediatric studies, and oral antibiotics were substituted in the other 11 studies [5]. The outcomes appear to be comparable with these two strategies. The only randomized controlled trial of oral antibiotics versus placebo found no advantages in using antibiotics [4]. This would suggest that undiagnosed partially treated bacterial infection is not common in these patients.

## Conclusion

About half of the pediatric oncology patients in our program had at least one admission for FN. Intravenous antibiotics were discontinued with an ANC ≤ 500/mm^3 ^in about half of episodes of FN, with oral antibiotics then being prescribed only for patients with proven bacterial infections. The few secondary bacterial infections occurred prior to discontinuation of IVAMT, and no patients required readmission because of suspected or proven infection. Based on this and previous studies, it is not necessary to routinely prescribe oral antibiotics in children who are discharged with an ANC ≤ 500/mm^3^. A prospective randomized study could determine if use of objective criteria in addition to clinical judgment could increase the number of children who can safely have IVAMT discontinued while still neutropenic.

## List of abbreviations

AMC – absolute monocyte count

ANC – absolute neutrophil count

APC – absolute phagocyte count

°C – degrees Celsius

FN – febrile neutropenia

IVAMT – intravenous antimicrobial therapy

MDI – microbiologically defined infection

## Competing interests

The author(s) declare that they have no competing interests.

## Authors' contributions

HH collected all data and wrote part of the manuscript. PEG helped with study design and reviewed the manuscript. JLR helped with study design and wrote part of the manuscript.

## Pre-publication history

The pre-publication history for this paper can be accessed here:


